# Acceptability, Safety, and Resonance of the Pilot Digital Suicide Prevention Campaign “Better Off With You”: Qualitative Study

**DOI:** 10.2196/23892

**Published:** 2021-03-03

**Authors:** Elise Rose Carrotte, Marianne Webb, Anna Flego, Bonnie Vincent, Jack Heath, Michelle Blanchard

**Affiliations:** 1 Anne Deveson Research Centre SANE Australia South Melbourne Australia; 2 Melbourne School of Psychological Sciences The University of Melbourne Melbourne Australia

**Keywords:** suicide, interpersonal theory of suicide, social media, co-design, lived experience

## Abstract

**Background:**

The Interpersonal Theory of Suicide posits that there are three key elements of suicidal behavior: perceived burdensomeness, thwarted belongingness, and the acquired capability for suicide. The digital campaign *Better Off With You* was developed to directly challenge the idea of perceived burdensomeness among people who are contemplating suicide in 2 communities within Australia.

**Objective:**

The aim of this study is to explore the needs and preferences of people with lived experience of suicidal thoughts and actions to inform the development of *Better Off With You.*

**Methods:**

This study involved a series of focus groups that aimed to discuss campaign messaging, scope, and approach. People with lived experience of suicidal thoughts and actions attended the focus groups. After the completion of initial focus groups, the results informed the creation of campaign collateral by creative agencies. Early versions of the campaign collateral were then presented in the user testing sessions. Transcriptions were analyzed via thematic analysis.

**Results:**

In total, 13 participants attended the focus groups and 14 attended the user testing sessions. The following three overarching themes were presented: *acceptability*, *safety*, and *resonance*. Participants believed that suicide is a serious and ongoing issue in their communities and welcomed a localized suicide prevention focus via peer-to-peer storytelling. The idea of perceived burdensomeness required clarification but was perceived as acceptable and relevant. Participants seemed drawn toward peer narratives that they perceived to be authentic, genuine, and believable as given by real people with lived experience. Campaign messaging needs to be clear and empathetic while directly talking about suicide. Participants did not anticipate any significant negative or harmful impact from any campaign videos and highlighted the importance of providing appropriate help-seeking information.

**Conclusions:**

This iterative study provided important insights and knowledge about peer-to-peer storytelling in suicide prevention campaigns. Future campaigns should involve simple messaging, be validating and empathetic, and consider including a lived experience perspective.

## Introduction

### Background

In 2016, there were approximately 800,000 deaths by suicide globally [1]. In Australia, approximately 2.1 million people aged 16 to 85 years have thought about taking their own lives at some point [2]. There are a number of theories that provide various insights into suicidal thoughts and behavior [3]. To understand antecedents to suicide, one of these key theories—the Interpersonal Theory of Suicide—posits that there are 3 key elements of suicidal behavior: perceived burdensomeness, thwarted belongingness, and the acquired capability for suicide [4]. Both perceived burdensomeness and thwarted belongingness are significantly correlated with suicidal thoughts and actions [5].

Digital platforms and social media are increasingly being used to drive health promotion campaigns [6,7]. Indeed, social media has a range of potential benefits for suicide prevention, further providing an anonymous, accessible, and nonjudgmental forum for sharing experiences [8]. However, only a few studies have reported on the development of a safe campaign using social media for suicide prevention purposes [8,9]. To date, only one known campaign has published findings with regard to this approach, which was focused on youth [10]. Critically, there is some concern that the misuse of social media could possibly lead to the risk of contagion [11], where suicidal behavior, thoughts, or deaths spread through a group or community. However, following appropriate media guidelines and designing campaigns with people with lived experience is likely to mitigate these risks [10] alongside appropriate social media moderation. In fact, aligned with the Papageno effect, media can play an important, protective role in sharing alternative approaches to suicide, such as through suicide prevention campaigns [12]. Suicide prevention may involve various messages, including encouraging those at risk of suicide to access the appropriate mental health support, connect with others, and challenge their suicidal thoughts to reduce morbidity and mortality [13-16].

### Better Off With You: A Suicide Prevention Initiative

In response to this need, *Better Off With You* was developed, a digital suicide prevention campaign underpinned by the Interpersonal Theory of Suicide [4]. The campaign specifically aims to challenge perceived burdensomeness: the common schema experienced by people contemplating suicide that they are a burden on their family, friends, and other people. Perceived burdensomeness may relate to many areas, including worry about being an emotional or financial burden on others. Such a schema may lead people to believe that their family, friends, and community are *better off without them* or that their death would be a relief to those around them. Perceived burdensomeness was chosen as a key focus of the campaign, as several reviews have identified statistically significant relationships between perceived burdensomeness, suicidal thoughts, and suicide attempts across a range of populations [5,17,18]. In fact, some studies have found perceived burdensomeness to be a stronger predictor of suicidal behavior than thwarted belongingness [18]. Furthermore, no known campaigns have focused on this schema, meaning that it is a novel focus for such a campaign.

Although digital suicide prevention campaigns currently exist in Australia (eg, R U OK?, #YouCanTalk campaigns, [19,20]), these campaigns generally target thwarted belongingness rather than perceived burdensomeness and typically focus on educating the community to reach out to those at suicide risk. In contrast, *Better Off With You* directly targets people who may be contemplating suicide. The campaign is delivered through video stories featuring people with lived experience of suicidal thoughts and actions, encouraging people to challenge their cognitions and visit a campaign website for more information about the campaign, burdensomeness, and help seeking [21]. This focus on people with lived experience is important, as previous evidence has found that peer-led interventions can significantly impact areas such as quality of life, decreasing intensity of suicidal thoughts, and improving a sense of community [22,23].

Better Off With You was developed in partnership with 2 primary health networks (PHNs), which are organizations funded by the Australian Government Department of Health to deliver an efficient and effective primary health care system, including commissioning community-based suicide prevention activities. The 2 PHN partners, Northern Sydney PHN and Northern Queensland PHN, selected targeted areas within their regions to undertake the campaign pilot: the Northern Beaches of Sydney and the Whitsunday, Isaac, and Mackay area of Northern Queensland. These areas were chosen based on the following criteria: areas of greatest need (based on suicide rates and community concern); existing local networks where suicide prevention action planning and community engagement were already underway (including collaboration with local hospital networks, service providers, emergency services, and other community groups); strong local coordination, including capacity of staff within the specific PHN area to be a key contact for supporting campaign implementation; and preparedness of local services to respond to people seeking help in crisis, including emergency department referral pathways and follow-up care after a suicide attempt.

The campaign was developed iteratively with a direct involvement from the local communities and integrating best practice media guidelines for suicide prevention–focused communications. This included having a targeted, clear message with an emphasis on hope [15,16]. In addition, the approach was designed to engage the local community and provide opportunities to be a part of the change, aligned with evidence-informed, community-led suicide prevention approaches [24].

### Objectives

The aim of this study is to explore the needs and preferences of people with lived experience of suicidal thoughts and actions to inform the development of a digital peer-to-peer suicide prevention campaign pilot, *Better Off With You,* targeting perceived burdensomeness in 2 Australian communities.

## Methods

### Design

This formative study was qualitative in nature and involved focus groups to discuss initial campaign messaging, scope, and approach. Focus groups were chosen so that participants could share and reflect on each other’s beliefs, opinions, and experiences in the local community. The results informed the creation of the campaign collateral by creative agencies. Early versions of the campaign collateral were then presented in user testing sessions and then further refined before the campaign launch and impact evaluation.

### Participants

Focus groups used criterion-based sampling [25]—in this case, people who have experienced suicidal thoughts and actions living within 2 PHN areas. As such, the research included individuals who were most likely to benefit from (or be impacted by) *Better Off With You*.

Targeted recruitment occurred through local health and community organizations that are already engaged in suicide prevention efforts. These organizations were contacted and asked to share a flyer with relevant contacts, such as local suicide prevention champions and advocates within businesses and community organizations. Potential participants were also contacted through social media advertisements. Participants were recontacted for user testing workshops alongside additional contact with local stakeholders and snowballing (asking the existing focus group participants to share a recruitment notice with their networks).

Participants were required to meet the following inclusion criteria: be aged 20 years or older, currently or previously resided in the area, have a relevant lived experience (identified through the yes or no question: *Have you ever experienced a personal crisis or mental health issues relating to suicide [that is, experienced suicidal thoughts or actions yourself]*) but felt well enough to discuss their experiences or speak about this topic in a workshop context, and had not attempted or seriously contemplated suicide in the last 12 months. The inclusion criterion related to lived experience was to ensure that the participants were able to comment on the campaign messaging and materials with respect to their own personal experiences, rather than speaking theoretically or from the perspective of a family member or friend. The inclusion criterion relating to age (≥20 years) was specifically chosen after a review of suicide rates among different demographics in the local communities. This review identified that people aged 20-60 years were particularly at risk and were thus an important target audience of the campaign.

### Ethical Considerations

The study was reviewed and approved by Human Research Ethics Committee, Bellberry Limited (2019-03-230). As the study was iterative, the latter stages of the study were reviewed via amendments to the original application. Research was conducted by a multidisciplinary team with expertise in mental health research, suicide prevention, public health, qualitative research methods, and psychology.

Participant safety was prioritized by incorporating strategies used in previous suicide-related research [9]. Participants prepared a wellness plan to support them if they experienced distress during the workshop. A local mental health clinician was present to support participants should they experience distress during the workshop. Participants were requested to follow *safe sharing guidelines* and not disclose the details of suicide attempts in the session (either their own or those that have occurred in their community). Furthermore, participants were provided with crisis service contact details before focus groups through email contact, consent forms, and in follow-up emails within 48 hours of participation.

### Measures and Procedure

Focus groups and user testing sessions were attended by 3 female members of the project team: MW and EC, researchers who cofacilitated the discussion and who have experience in qualitative data collection and analysis, and BV, project lead, who provided oversight and support during discussion and took field notes. Sessions ran for 1.5-2 hours and were conducted in spaces such as local clinic meeting rooms and community centers.

Each session commenced with participants completing consent forms and an ice breaker. Each session was audio-recorded, and the participants were reimbursed with a Aus $50 (US $38) gift voucher. Participants were asked to complete feedback forms covering participants’ perceptions of the session’s safety at the end of each session, whether the session was interesting, whether they felt heard during the session, or whether they felt *Better Off With You* could make a difference in their community. Each of these was rated on a 5-point Likert scale from strongly disagree to strongly agree. Participants were also asked if they were interested in being recontacted for user testing sessions in the coming months.

Content was informed by a discussion guide. Focus groups involved an in-depth exploration of individual experiences, needs, and preferences related to content, format, and channels to assist in designing *Better Off With You*. Discussion questions pertained to memorable health campaigns, important messages around suicide prevention, discussion around perceived burdensomeness and its relevance, and format of the campaign. Region-specific questions were asked, including questions about the impact of suicide on their local community and the types of imagery or messaging that would appeal to the community.

User testing sessions were held around 3 months after the workshops. These involved reviewing the near-final campaign materials to ensure that they met the design requirements identified in the focus groups. Participants were shown 30-second and 3-min versions of the campaign videos featuring local people with lived experience of suicidal thoughts and actions and filmed in local settings. There were 3 video stories per region; each 30-second version was shown and discussed, and one 3-min version was shown and discussed. Discussion questions pertained to immediate reactions to the videos, likes and dislikes, perceived messages in the videos, and perceived appropriateness for the local community. Participants also discussed website design and architecture (*wireframes*).

### Data Analysis

Descriptive statistics were calculated for written feedback forms (percentage agreement and means or SDs). Audio files were transcribed verbatim. Transcriptions, field notes, and written feedback were analyzed via thematic analysis using the methodology outlined by Braun and Clarke [26] using NVivo 12 (QSR International). This was a recursive process that was both deductive and inductive in nature. The initial steps involved immersion in the data by 2 authors (MW and EC), via reading, rereading, and listening to recordings. These authors then independently coded each transcript line by line, and met to discuss and generate overarching themes based on patterns identified between the codes. Latter transcripts were then coded using these initial themes as a guide. The authors regularly met to discuss the thematic analysis and to modify and refine the themes, subthemes, and codes.

## Results

### Overview

The results are presented chronologically below, with each subphase structured according to 3 themes: *acceptability*, *safety*, and *resonance*. These overarching themes were applied on both the focus groups and user testing sessions and are discussed in relevance to each iterative stage. Subthemes, including *clarity of messaging* and *balancing hope and realism*, were discussed where appropriate. Descriptive statistics for feedback forms are presented in Multimedia Appendix 1.

### Focus Groups

In total, 13 people with lived experience of suicidal thoughts and actions participated in focus groups in June 2019. The first workshop was held in Northern Queensland and included 6 women aged 27 to 59 years (mean 48.7, SD 11.6). Owing to recruitment difficulties, a second workshop could not be held in this PHN area; instead, 2 individual interviews were conducted with 2 women aged 48 and 64 years. A second workshop was held a fortnight later in New South Wales. This workshop included 3 women and 2 men aged 27-60 years (mean 44.8, SD 19.2). For workshop participants, written feedback (Multimedia Appendix 1) indicated a very high agreement (>90%, 10-11/11) that the workshop was safe, interesting, participants’ voices were heard, and the campaign could make a difference in the local communities.

#### Acceptability

Participants believed that suicide is a serious, ongoing issue in their communities and welcomed a localized suicide prevention focus. However, most participants did not immediately identify perceived burdensomeness as an important schema for someone contemplating suicide. Participants also had difficulty understanding the tagline *Better Off With You* and its relationship to burden without context. Participants tended to initially conceptualize the message as being said by other people (family or friend) to a suicidal person, rather than being said by people with a lived experience. They felt that this perspective would have a negative impact on someone contemplating suicide and that they would feel guilty and ashamed of having suicidal thoughts:

I just got the feeling of guilt [from the tagline “Better Off With You”]…that it’s not about the person who is suicidal, it’s about the other people; we are better off with you. We’re not focusing on the person and trying to find out what’s going on for you, and that goes into that guilt tripping: “We want you here so don’t you dare kill yourself”.

Female participant, New South Wales session

Interpretation of the tagline was discussed in detail; hence, the first subtheme was identified: *clarity of messaging*. Participants found the tagline to be clearer once they understood the broader messaging of the campaign. Once participants understood that the message would be delivered by someone with a lived experience rather than by a third party and that the message was free from judgment or shame, they generally accepted the tagline. Similarly, participants accepted targeting perceived burdensomeness once this was explained clearly and contextualized, even though participants might not have experienced this schema as a part of their own lived experience with suicidal thoughts and actions:

I think [the message is] actually very, very powerful. I think the idea of if you can just get yourself over this point – and there is a point and it is going to change. It may not get better straight away, but if you can just get yourself over this hurdle and out of this rut again maybe you can start helping yourself a little bit.

Female participant, Queensland session

#### Safety

Participants did not identify any component of the campaign that was identified as inherently triggering or unsafe, as long as the messaging was clear and considered. However, they spoke to the nuance of maximizing emotional safety in the campaign—that is, minimizing distress, avoiding critical incidents, and ensuring viewers feel comfortable with the campaign.

The importance of validating people’s lived experience through the narratives was highlighted, with participants noting that being suicidal “is a big deal and it is hard” (female participant, New South Wales session). They warned against trivializing experiences with presumptive messaging, such as *you will get over it.*

Participants reported the importance of including help-seeking information as a part of the campaign in the event that a viewer of the campaign becomes distressed or triggered (ie, re-experiencing a traumatic event): “I think if you’ve going to have a powerful ad like that, at the end you need to have something about if it triggered you, here’s the number to call” (female participant, Queensland session). They highlighted the importance of including a range of local, national, and web-based services, including 24/7 services. They reported that the website accompanying the campaign should also include support for carers, families, or friends. Participants also reported that help seeking can be challenging; they suggested encouraging campaign viewers to keep trying even if they did not find the right support immediately.

#### Resonance

Real stories were perceived as likely to resonate with the campaign’s target audience. Participants identified that an authentic and honest tone would have a real impact through voices of those with lived experience of suicidal thoughts and actions and who have walked the path themselves (rather than actors or celebrities):

It’s actually using people who have been through it and have survived and have turned their lives around...there is nothing worse than listening to some paid actor and it’s like “you have no idea…you haven’t been in my shoes. I can’t have any faith in what you’re telling me because you’re not even real.”

Female participant, Queensland session

It was identified that the campaign was most likely to resonate if the message was simple, without being too simplistic, and did not trivialize experiences; participants highlighted the importance of acknowledging the complexity of issues and of people’s lives.

Participants reported that for the campaign to resonate, it must be engaging. Participants recommended that the campaign should have a serious but hopeful tone and use conversational language rather than formal language, which might ostracize some viewers. They identified that the video content should be brief and eye catching to hold the audience’s attention, should not be overproduced (ie, should not include overly dramatic music), and should talk directly about suicide rather than just alluding to it.

Generally, participants were enthusiastic about the campaign being localized. They reported that known geographic locations and landmarks could be shown to be locally identifiable. Participants also highlighted the importance of diverse representations in the campaign, thereby targeting a range of audiences, such as people from farming or mining communities, young people who are not engaged in popular local social activities or cultures such as surfing and sport, and people from culturally and linguistically diverse backgrounds.

### User Testing

**Figure 1:**
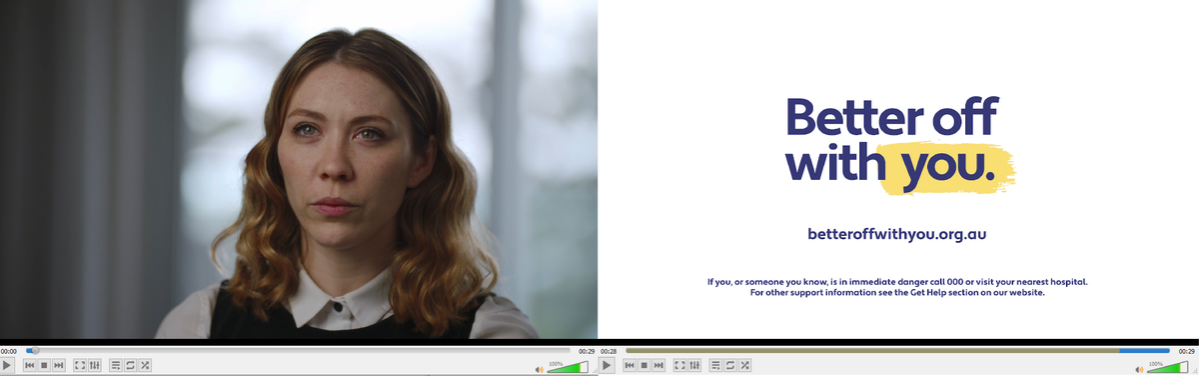
Screenshots of draft Better Off With You videos (video 1/6; Phoebe’s story; 30-second version) discussed in user testing sessions.

**Figure 2:**
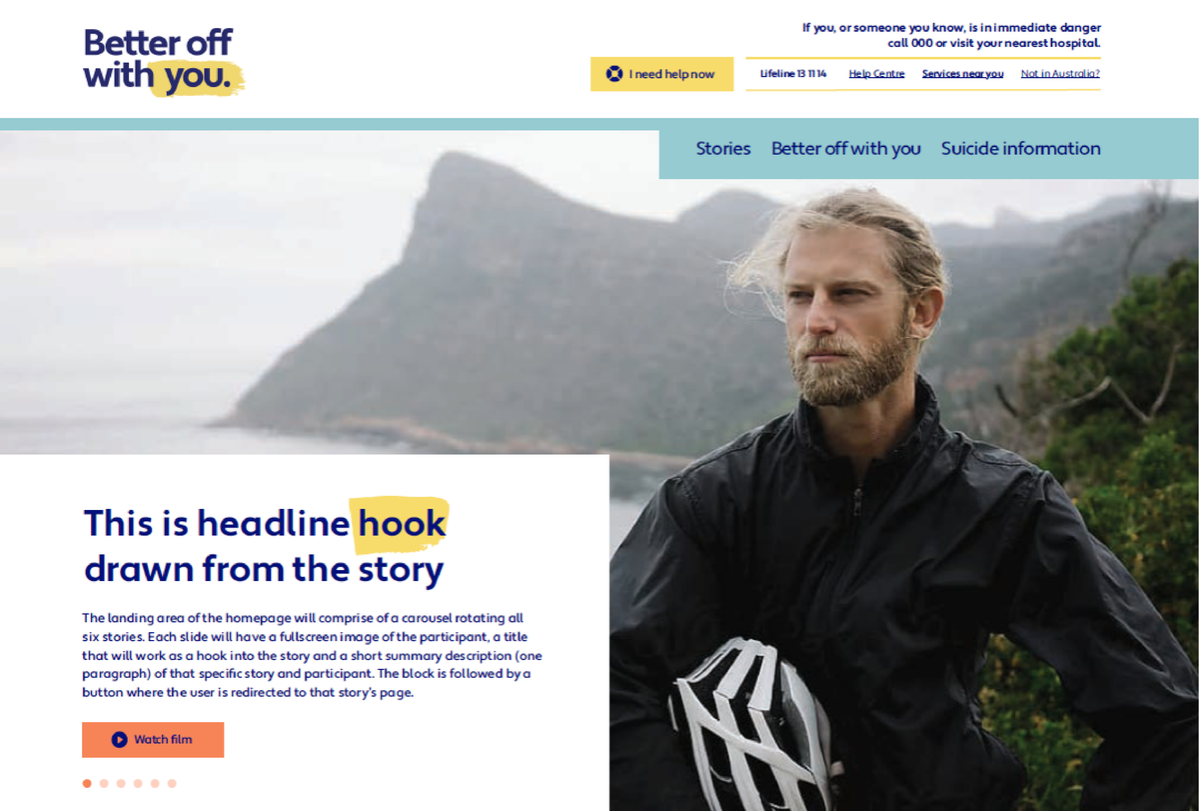
Draft Better Off With You website landing page design discussed in user testing sessions.

The focus group findings informed the development of the campaign collateral while considering tone, language, length of videos, clarity of messaging, and opportunities for dissemination. Draft collateral was presented to participants in user testing sessions (examples are presented in Figures 1 and 2). Videos featured a person with personal experience of suicidal thoughts and actions discussing events, emotions, and thought processes that preceded or contributed to their suicidal thoughts, how they coped, and what life has been like since the crisis had passed. Thoughts around perceived burdensomeness were highlighted in each story. In each video, the storyteller spoke directly to the camera, with local overlay footage edited throughout the video (eg, the participant walking along the beach, spending time with friends, or walking their dog). The final shot of each video was the *Better Off With You* logo, website link, and crisis support numbers. A 30-second teaser version was produced for each story for social media, designed to draw in the viewer and then direct the viewer to the *Better Off With You* website to watch the longer 3-min version of the video and access support information.

A total of 3 user testing sessions occurred across September to October 2019: 2 in New South Wales and 1 in Northern Queensland. In total, 14 participants attended these sessions. A total of 10 participants attended in New South Wales, comprising 4 men and 6 women aged 23-68 years (mean 42.2, SD 15.1); 4 participants participated in Northern Queensland, with 1 man and 3 women aged 43-64 years (mean 51.5, SD 9.0). Written feedback (Multimedia Appendix 1) indicated a very high agreement (14/14, 100%) that the workshop was safe and interesting, and participants’ voices were heard. Feedback was mixed for the items pertaining to the campaign materials, with the strongest agreement for the videos being engaging (12/14, 86%), likely to encourage help seeking (14/14, 100%), and likely to give viewers a sense of hope for the future (13/14, 93%). The lowest percentage agreement was for the item: *the campaign could distress viewers*, with only 29% (4/14) agreement.

#### Acceptability

Messaging was frequently discussed, both prompted and unprompted by the facilitators. As seen in the focus groups, participants accepted the general message behind *Better Off With You*. The message was generally perceived as fairly clear in the 30-second videos but became diluted during the longer 3-min videos, which involved more of the storyteller’s narrative. Participants identified key (but unintended) messages they noticed in the longer videos, which included *there is hope*, *find the right diagnosis*, and *talk about it*. These were not necessarily received as inappropriate messages for a suicide prevention campaign but were not the intended primary focus of *Better Off With You*. Participants felt that the *you are not a burden* message often came through as secondary to these other messages in the 3-min videos, was too subtle, or was lost in the narrative:

I found the 30 seconds captured me, whereas this...it was just your classic sort of suicide video. Because I loved the message so much about this burden on family, and it was only really mentioned very briefly...

Female participant, New South Wales session

Participants were most accepting of the 30-second videos, generally identifying them as engaging, real, and honest. These clips were very rapid, and some participants commented that they could be simplified slightly to improve comprehension. Regardless, they reported that the message in these short videos was clear and strong. Participants often identified a sense of hope in these videos and praised this tone, for example: “(The video) showed the future, from a dark place...it showed a possible future” (female participant, Queensland session).

Feedback around the acceptability of the longer, 3-minute versions of the videos was more mixed. Although some participants identified these as powerful, “open, and honest” (male participant, New South Wales), others reported that the videos were not as engaging or clear. Creative flow significantly impacted this result, particularly the choice of music and rapid editing of overlay or dialog, which were seen as disjointed, distracting, or inappropriate to the topic at times. Suggestions were made to simplify the editing and alter the music to ensure that the narrative was engaging and the message was clearer.

The website design was generally viewed favorably. Although some felt the design was “a bit clinical” (female participant, Northern Queensland session), the graphic design was generally well received. Participants recommended that the website content have a personal, informal, and direct tone:

It’s a clean website; it’s not cluttered. And it seems to be easy to navigate, and the colour scheme is the same. So I think it’s a good one.

Female participant, New South Wales session

#### Safety

Participants did not anticipate any significant negative impact from any campaign videos. A few participants experienced an emotional response to the videos within the sessions, but no participants identified being emotionally triggered or feeling unsafe after seeing the campaign materials.

Participants identified that help-seeking messages in the videos were necessary to ensure that viewers knew where to seek help if they were experiencing suicidal thoughts. However, specific descriptions of help seeking from the storytellers often brought about comparative responses. A second subtheme was identified through this discussion: *balancing hope and realism*. Participants spoke to the challenge of needing to balance the message of *seek help if you are feeling suicidal* with a realistic assessment of the challenges associated with seeking help in local mental health services. If participants interpreted the storyteller’s help-seeking journey as being a straightforward process, then this was met with rejection:

The fact that [the storyteller] said, “You can actually talk to your doctor and you won’t be judged,” but there will be people out there who say, “Well, how would you know? My doctor judged me badly and I’m never going back to this doctor.”

Female participant, New South Wales session

Many participants identified that they had personally experienced challenges while seeking help for mental health or suicidal thoughts; however, focusing too much on this in the videos could be seen as off-putting and detracting from the intended messages of the campaign. This meant that if participants interpreted the storyteller as being privileged with access to a range of health care professionals and self-care resources, then this interpretation could also be taken in the wrong way:

I feel [the message around help seeking] is a little bit risky... It’s like, just keep on trying and maybe you’ll get there, and you’ve got to do ballet and see a psychiatrist and see a psychologist and go to the gym, stay away from the screens and ask someone to help you... And even when you do all that, you might not click with the right person.

Female participant, New South Wales session

Participants highlighted the need for the website to be easy to navigate, particularly in the event that someone accesses the website in a state of distress or suicidal crisis. They suggested a clear signposting of *Better Off With You* as a suicide prevention initiative, including more direct information about managing thoughts around burdensomeness on the website:

Just having something like some practical skills, like how to talk to someone about the burden...so just some questions you could ask. Because that’s really what it’s about, isn’t it? Like there’s all different layers of help, but if you do feel that you are a burden to others, just being able to sit down, say with a mother, and say, “These are the feelings I’m having,” and what questions can you ask.

Female participant, Queensland session

#### Resonance

Participants seemed drawn to storytellers who were perceived to be authentic, genuine, and honest. The sense of authenticity was enhanced by seeing storytellers express clear emotions (eg, becoming teary when discussing a difficult experience), ensuring that the camera was on the storyteller’s face when they were discussing sensitive elements of their stories, and showing storytellers in their own environments. This contributed to some storytellers being viewed as engaging, likable, and *quite authentic* (male participant, New South Wales session).

Participants also commented on the relatability of the participants’ stories. Some participants reported relating to the elements of the storytellers’ narratives, such as their thought processes. Relatability was enhanced because of some storytellers who do not look like they fit the stereotypes of people experiencing mental health issues or suicidal thoughts, for example: “I think she (the storyteller) looked like someone who you would not imagine would struggle” (male participant, New South Wales session).

Points of contention were identified in the longer videos, which explored more of the storyteller’s journey. Aligned with the subtheme of *balancing hope and realism* discussed above, when more specifics of a diagnosis or recovery journey were provided, this tended to invoke a more comparison-based response from the participants, who were likely to comment along the lines of *not everyone can access that support* or *in my experience that did not work*. Similarly, if participants mentioned specific symptoms of mental health issues (such as mania if they experienced bipolar disorder), then the participants tended to find this less relatable if they did not reflect their experience. The more specific details provided, the more likely participants were to focus on the details and lose a sense of the overall intended messages due to perceived irrelevance or being *invalidating* (male participant, New South Wales). Participants tended to respond critically to the instances of advice-giving or blanket reassurance provided by the storytellers:

I think for young people [the message] might be a bit too strong...Kind of like a dad saying, “You’re not thinking straight if you’re thinking about killing yourself.” And it’s like, “Well, what would you know?”

Male participant, New South Wales session

## Discussion

### Principal Findings

This qualitative, iterative study explored the needs and preferences of people with lived experience of suicidal thoughts and actions for a digital peer-to-peer suicide prevention campaign pilot, *Better Off With You*. We conducted a series of focus groups to discuss initial campaign messaging and approaches. On the basis of this feedback, campaign collateral was then developed before being presented in user testing sessions with local participants with lived experience for further feedback and recommendations. To the best of our knowledge, this is the first study to report on the design and development of a digital suicide prevention campaign focusing on perceived burdensomeness.

The findings of this study highlight the challenges in developing a suicide prevention campaign that successfully conveys a clear, simple message. The intended burdensomeness message did not immediately resonate with participants, with participants also perceiving a variety of other appropriate, positive campaign messages. This was important, as participants highlighted the need for a hopeful tone. Concerns were also raised about the key message being misinterpreted. Even though research has shown a strong correlation between perceived burdensomeness and suicidal behavior [18], there may be a low awareness in the general public about this relationship. Given that we have not found any other suicide prevention campaigns that focused on perceived burdensomeness, this finding is not surprising. It reinforces the need to have ongoing end user involvement in the development of any suicide prevention campaign to ensure that the intended message is understood by the target audience and communicated in a clear, validating manner. It also highlights the potential role of the media in conveying a sense of hope and alternative pathways in the context of suicidal thoughts and actions.

A major finding of the study was the participants’ strong preference to include first-person perspectives in the campaign. This resonance is aligned with a previous research study that suggests that peer-to-peer mental health and suicide programs with relatable, credible lived experience perspectives are an effective way to decrease stigma and increase help seeking [27]. It also reflects a recent research study by Thorn et al [10], who found that young people wanted to see real people in videos and photographs for a social media suicide prevention campaign, rather than professional actors or models. Not only was the peer-to-peer approach seen to be an authentic and acceptable, participants felt that a campaign where the message was delivered by another person, such as a family member or friend, would not be as powerful. Our findings contribute new insights and knowledge about the design elements required for developing effective lived experience suicide prevention videos, as shown in recent studies reporting on the effectiveness of videos targeting farmers [28] and lesbian, gay, bisexual, transgender, intersex, and queer communities [29]. These findings also suggest that a third-person approach, while well intentioned, may not engage the target audience of those contemplating suicide effectively.

Regardless, the study identified the importance of ensuring that these real stories are perceived to be realistic and relatable while acknowledging the inequities of access to appropriate support. For example, participants were critical of the stories they perceived to be downplaying the realities of seeking help for suicidal thoughts and actions; however, they agreed that help-seeking messages were important. This result highlights the need to strike a balance between being true to a person’s lived experience while ensuring that their story is told safely and realistically.

Ensuring the material does not have any negative or unexpected impact is critical for any suicide prevention intervention, particularly for campaigns intended for a mass media distribution across a region or community. The findings of this study suggest that it is possible to develop a safe suicide prevention campaign that addresses perceived burdensomeness. Although there are existing evidence-based guidelines for developing suicide prevention campaigns [15,16], this study provides new insights into the nuances required to develop safe suicide prevention messages without alienating the target audience; for example, acknowledging the challenges of seeking help, without discouraging people or suggesting that there is only one *right* way to manage suicidal thoughts. Furthermore, the written feedback and lack of adverse incidents indicate that there were no negative or unexpected impacts on participants because of participating in the study or seeing the draft campaign materials. This supports research indicating that people with lived experience of suicidal thoughts and actions can be safely involved in this type of research [10,30,31].

### Research Translation

Findings from this study were communicated to the creative agencies involved in designing the website, video stories, and other campaign collaterals. Edits included changing dialog (including removing some lines that were highlighted by participants as invalidating or challenging), slowing down the pace, removing some of the overlay to simplify the videos, and replacing some of the music cues. Website changes included a clearer branding of the website as a suicide prevention initiative, adding more information about perceived burdensomeness, and various language changes. A future publication will report on the effectiveness of this suicide prevention campaign pilot when delivered via social media.

### Limitations

This study has some limitations. As a small pilot conducted in only 2 regions of Australia, the generalizability of the findings may be limited. Notably, each workshop ranged between only 4 to 6 participants. Difficulty with recruitment is common in suicide-related research [9], and it was difficult to find people in the community who fit the inclusion criteria and were willing and able to participate within a short recruitment timeline. Recruitment was restricted to specific geological areas and budget or overarching project timeline constraints, likely impacting upon the participation rates.

Younger participants and men were underrepresented, which is not unusual in health research [32]. However, with an increased buy-in from local stakeholders to help target local men more directly, we were able to improve the gender ratio for the user testing sessions, with 36% (5/14) being male participants. Future research should expand on this work to improve the generalizability to the specific needs of populations who are overrepresented in suicide statistics, including men, Aboriginal and Torres Strait Islanders, those from cultural and linguistically diverse backgrounds, and those with diverse sexual and gender identities.

Despite the small sample and recruitment challenges, the feedback was rich. Indeed, given the sensitive nature of the topic, a smaller and more intimate size may have facilitated a more meaningful contribution in each session with the opportunity to discuss ideas in greater depth in smaller groups.

Finally, it is possible that using direct payment incentivized participants to sign up for the study who may not have had sufficient experience or local expertise; however, considering the depth of the feedback, the authors do not believe this impacted upon the quality of the data.

### Conclusions

This iterative study provided important new insights and knowledge about how the contribution of people with lived experience of suicidal thoughts and actions is essential for the development of an acceptable, safe, and resonant suicide prevention campaign. The messaging must be clear, simple, and validating, and the inclusion of a lived experience perspective is particularly valuable. This feedback was critical for developing the *Better Off With You* pilot*,* which has now been rolled out. Subsequently, opportunities for expanding the campaign are being explored. Impact evaluation will be discussed in a future publication.

## Appendix

Multimedia Appendix 1 Feedback form summary.

## Abbreviations

PHN: primary health network
