# Supplementary material for: Acceptability, Safety, and Resonance of the Pilot Digital Suicide Prevention Campaign “Better Off With You”: Qualitative Study
Source: JMIR Form Res. 2021 Mar 3;5(3):e23892. doi: 10.2196/23892 (PMC7970163; doi:10.2196/23892)
Supplement: Multimedia Appendix 1 [file formative_v5i3e23892_app1.docx]

**Multimedia Appendix 1.** Feedback form summary.

**Table 1. Summary of feedback forms from focus groups.**

| **Question** | **Strongly disagree (1)** | **Disagree (2)** | **Neither Agree or Disagree (3)** | **Agree (4)** | **Strongly agree (5)** | **Mean /5** | ***SD*** |
| --- | --- | --- | --- | --- | --- | --- | --- |
|  |  |  |  |  |  |  |  |
| I felt that my voice was heard during the workshop | 0 | 0 | 0 | 1 (9%) | 10 (91%) | 4.9 | 0.3 |
| I felt safe during today's workshop | 0 | 0 | 0 | 1 (9) | 10 (91%) | 4.9 | 0.3 |
| The workshop was interesting | 0 | 0 | 1 (9%) | 3 (27%) | 7 (64%) | 4.5 | 0.7 |
| The BOWY campaign pilot sounds like it could make a difference in my community | 0 | 0 | 1 9%) | 3 (27%) | 7 (64%) | 4.5 | 0.7 |

*N*=11. Two participants provided input via 1:1 interviews and as such did not complete feedback forms.

**Table 1. Summary of feedback forms from user testing sessions.**

| **Question** | **Strongly disagree (1)** | **Disagree (2)** | **Neither Agree or Disagree (3)** | **Agree (4)** | **Strongly agree (5)** | **Mean /5** | ***SD*** |
| --- | --- | --- | --- | --- | --- | --- | --- |
|  |  |  |  |  |  |  |  |
| I felt that my voice was heard during the session | 0 | 0 | 0 | 0 | 14 (100%) | 5.0 | 0.0 |
| I felt safe during today's session | 0 | 0 | 0 | 0 | 14 (100%) | 5.0 | 0.0 |
| The session was interesting | 0 | 0 | 0 | 1 (7%) | 13 (93%) | 4.9 | 0.3 |
| The BOWY campaign pilot sounds like it could make a difference in my community | 0 | 0 | 0 | 5 (36%) | 8 (57%) | 4.6 | 0.5 |
| The BOWY videos are engaging | 0 | 0 | 1 (7%) | 6 (43%) | 6 (43%) | 4.4 | 0.7 |
| The BOWY campaign has a clear message | 0 | 1 (7%) | 2 (14%) | 4 (29%) | 7 (50%) | 4.2 | 1.0 |
| The BOWY campaign could challenge viewers’ perceptions of being a burden | 0 | 1 (7%) | 1 (7%) | 9 (64%) | 3 (21%) | 4.0 | 0.8 |
| The BOWY campaign could encourage help-seeking | 0 | 0 | 0 | 9 (64%) | 5 (36%) | 4.4 | 0.5 |
| The BOWY campaign could distress viewers | 1 (7%) | 2 (14%) | 7 (50%) | 4 (29%) | 0 | 3.0 | 0.9 |
| The BOWY campaign could give viewers a sense of hope for the future | 0 | 0 | 1 (7%) | 7 (50%) | 6 (43%) | 4.4 | 0.6 |

*N*=14. Rows may not add up to 100% if participant skipped a question.
